# Cohort Profile: Centro de Integração de Dados e Conhecimentos para Saúde (CIDACS) Birth Cohort

**DOI:** 10.1093/ije/dyaa255

**Published:** 2020-12-30

**Authors:** Enny S Paixao, Luciana L Cardim, Ila Rocha Falcao, Naiá Ortelan, Natanael de Jesus Silva, Aline dos Santos Rocha, Samila Sena, Daniela Almeida, Dandara Oliveira Ramos, Flávia Jôse Oliveira Alves, Nívea Bispo, Sanni Ali, Rosemeire Fiaccone, Moreno Rodrigues, Liam Smeeth, Elizabeth B Brickley, Liliana Cabral, Carlos Teles, Maria Conceição N Costa, Maria Yury Ichihara, Mauricio L Barreto, Rita de Cássia Ribeiro Silva, Maria Gloria Teixeira

**Affiliations:** 1 Centro de Integração de Dados e Conhecimentos para Saúde, Fiocruz, Salvador, Bahia, Brazil; 2 Epidemiology and Population Health, London School of Hygiene and Tropical Medicine, London, UK; 3 Escola de Nutrição, Universidade Federal da Bahia, Salvador, Brazil; 4 Instituto de Saúde Coletiva, Universidade Federal da Bahia, Salvador, Bahia, Brazil; 5 Departamento de Estatística, Universidade Federal da Bahia, Salvador, Bahia, Brazil

## Why was the cohort set up?

The CIDACS (Centre for Data and Knowledge Integration for Health) Birth Cohort is a Brazilian population-based cohort derived from linked data, developed to investigate the relationships between prenatal and early life events on health-related outcomes for infants, children, adolescents and mothers in the context of social inequalities. Societal determinants influence the health and well-being of the mother, the intrauterine environment and the fetal development before birth, increasing the risk of fetal loss as well as low birthweight and prematurity.^1^^,^^2^ Adverse birth outcomes, such as low birthweight, not only affect mortality and development during the early years, but also educational attainment, economic participation and long-term health outcomes.^3–6^ There is clear evidence that disruption (either biological or social) in the intrauterine life and/or during the first years of life can have significant consequences for health in adulthood, increasing the risk of diseases such as cardiovascular diseases, diabetes, psychiatric illness and even some cancers. Besides, lack of access to or poor quality of prenatal care has been associated with congenital malformations and rare late complications such as autoimmune diseases, diabetes and mental disorders.^7–15^

The design of CIDACS Birth Cohort follows a life course perspective, using routinely collected data from Brazil. The overall objective is to research the effect of obstetric and prenatal conditions, congenital infections and other potential social and environmental determinants and the impact of social policies on birth, growth, morbidity and survival, overall and in subgroups of interest in a dynamic Brazilian birth cohort. The use of large, routinely collected, high-quality social and health databases provides a unique opportunity to examine factors that might result in long-term and rare child and mother outcomes over time without the limitations of a traditional cohort, such as limited sample sizes and expensive resources.

The CIDACS Birth Cohort is housed at the Centre of Data and Knowledge Integration for Health (CIDACS), a unit of the Oswaldo Cruz Foundation in Bahia, Brazil. CIDACS also houses the 100 Million Brazilian Cohort. CIDACS works in the spectrum of data acquisition for linkage data from large Brazilian national databases, management, analysis and interpretation with ethical use and privacy issues.^16^

Ethical approval was obtained from the Federal University of Bahia’s Institute of Public Health Ethics Committee (CAAE registration number: 18022319.4.0000.5030).

## Who is in the cohort?

Brazil has about 3 million births a year. A total of 44 485 267 births were recorded in the live birth system (SINASC) over 2001–15. The CIDACS Birth Cohort population is composed of 24 695 617 (55%) children born alive in Brazil between 1 January 2001 and 31 December 2015 which linked with the baseline of the 100 Million Brazilian Cohort through common maternal information, which exists in the two datasets. All children with information recorded in the live birth system (SINASC) were eligible for linkage.

The SINASC records live births in Brazil, using a standardized form, completed by a health professional who assisted the child’s delivery. This form has information on pregnancy and delivery of newborns, including congenital anomalies, birthweight and sex. An evaluation of the birth registration system in Brazil found that over 97% of Brazilian live births are registered in this system.^17^^,^^18^

The baseline of the 100 Million Brazilian Cohort was created using administrative records from over 114 million individuals aged 16 years or older, whose families applied for social assistance via the Unified Register for Social Programmes (Cadastro Único para Programas Sociais: CadUnico). Since 2003, the CadUnico has become the main instrument used by the Brazilian government to assess the inclusion criteria of potential beneficiaries of social programmes. To be enrolled in CadUnico, one person in the family must provide information and required documents of all family members to an interviewer. This person must be at least 16 years old and, preferably, be a woman. The information is renewed periodically as long as the person is a candidate or enrolled in any one of the Brazilian benefits, such as Bolsa Familia (cash transfer for low-income families) and Minha Casa Minha Vida and Beneficio de Prestação Continuada (continuous benefit for people with long-term disability) among others.^19^ By the end of 2015, 40 542 929 families (comprising 114 001 661 individuals) had registered in CadUnico.

The characteristics of mothers and children in the CIDACS Birth Cohort were compared with the characteristics of the non-linked population of mothers and children registered in SINASC, to assess differences and similarities between our cohort populations (Table 1). A higher proportion of mothers of children born in the CIDACS Birth Cohort are younger, i.e. less than 20 years old (25% vs 15%) and unmarried (58% vs 43%), than those in the non-linked population recorded in SINASC. The proportion of mothers with 8 years or more of schooling were higher in the non-linked population (69%) compared with those included in the cohort (52%). Children included in the cohort were more likely to be born via vaginal delivery (60%) than the non-linked Brazilian births (42%). Children from minority ethnic backgrounds were included in the cohort. To date, the cohort includes 83 413 Indigenous children and 37 441 children born in Quilombo communities descended from African Brazilian fugitive slaves.

**Table 1.** Characteristics recorded in SINASC comparing the non-linked population with those included in CIDACS birth cohort

Characteristics	Births outside CIDACS cohort		CIDACS birth cohort	
	*n*	(%)	*n*	(%)
Child sex				
Male	10 129 574	51.22	12 648 797	51.25
Female	9 647 372	48.78	12 032 213	48.75
Missing/inconsistent	12 704	0.06	14 607	0.06
Birthweight (g)				
<2499	1 634 248	8.29	2 043 165	8.30
2500–6999	18 074 943	91.71	22 562 464	91.70
Missing/inconsistent	80 459	0.41	89 988	0.36
Apgar score at 5 min				
<7	266 714	1.41	376 650	1.63
7–10	18 621 036	98.59	22 714 004	98.37
Missing/inconsistent	901 900	4.56	1 604 963	6.50
Congenital anomaly				
Yes	134 961	0.73	165 000	0.71
No	18 387 310	99.27	23 200 675	99.29
Missing/inconsistent	1 267 379	6.41	1 329 942	5.39
Maternal age				
8–20 years	3 010 017	15.23	6 159 307	24.95
20–34 years	14 225 523	71.99	16 499 140	66.84
35–49 years	2 524 681	12.78	2 024 292	8.20
Missing/inconsistent	29 429	0.15	12 878	0.05
Marital status				
Single/widow/divorced	8 361 271	42.95	14 072 538	58.02
Married/union	11 104 701	57.05	10 181 772	41.98
Missing/inconsistent	323 678	1.64	441 307	1.79
Maternal education				
None	323 204	1.67	530 371	2.20
1–3 years	1 264 181	6.55	2 418 072	10.04
4–7 years	4 353 070	22.55	8 558 396	35.54
≥8 years	13 360 026	69.22	12 574 177	52.22
Missing/inconsistent	489 169	2.47	614 601	2.49
Abortion or fetal loss				
Yes	1 989 121	11.94	2 953 632	14.38
No	14 663 689	88.06	17 587 394	85.62
Missing/inconsistent	3 136 840	15.85	4 154 591	16.82
Number of live children				
0 children	7 877 634	43.83	7 994 779	35.54
1–5 children	9 842 013	54.75	13 902 551	61.80
6 children	255 260	1.42	597412	2.66
Missing/inconsistent	1 814 743	9.17	2 200 875	8.91
Number of babies				
Singleton	19 360 422	98.01	24 186 064	98.10
Twins or more	393 849	1.99	468 004	1.90
Missing/inconsistent	35 379	0.18	41 549	0.17
Numbers of prenatal visits				
None	459 420	2.36	708 138	2.91
1–3 times	1 277 456	6.57	2 404 920	9.87
4–6 times	5 043 757	25.93	8 734 269	35.85
≥ 7 times	12 667 871	65.14	12 513 484	51.37
Missing/inconsistent	341 146	1.72	334 806	1.36
Method of delivery				
Vaginal	8 341 386	42.25	14 668 584	59.52
Caesarean section	11 400 142	57.75	9 975 810	40.48
Missing/inconsistent	48 122	0.24	51 223	0.21
Gestational age at delivery (weeks)				
≤27	90 090	0.46	108 903	0.45
28–31	159 315	0.82	201 834	0.83
32–36	1 337 572	6.86	1 694 243	7.01
37–41	17 670 759	90.62	21 630 250	89.45
≥42	241 716	1.24	546 364	2.26
Missing/inconsistent	290 198	1.47	514 023	2.08

SINASC, Sistema de Informação sobre Nascidos Vivos/Information System of Live Birth; CIDACS, Centre of Data and Knowledge Integration for Health.

### The linkage processes

We linked SINASC live births records with the baseline of the 100 Million Brazilian Cohort using the name of the mother, maternal age at birth, maternal date of birth and the municipality of residence of the mother at the time of delivery. We excluded records with missing or implausible names and duplicates. The linkage was performed with CIDACS-RL (Centre for Data and Knowledge Integration for Health- Record Linkage),^20^ a novel record linkage tool developed to link big administrative datasets at the CIDACS. The linkage is detailed described in Almeida *et al*. (2020).^21^

At CIDACS, the processing and linking of identified databases follow legal frameworks related to ethics, privacy and data security. The study protocol was reviewed and approved by the Federal University of Bahia’s Institute of Public Health Ethics Committee (CAAE registration number: 18022319.4.0000.5030).

## How often have they been followed up?

**Figure 1:**
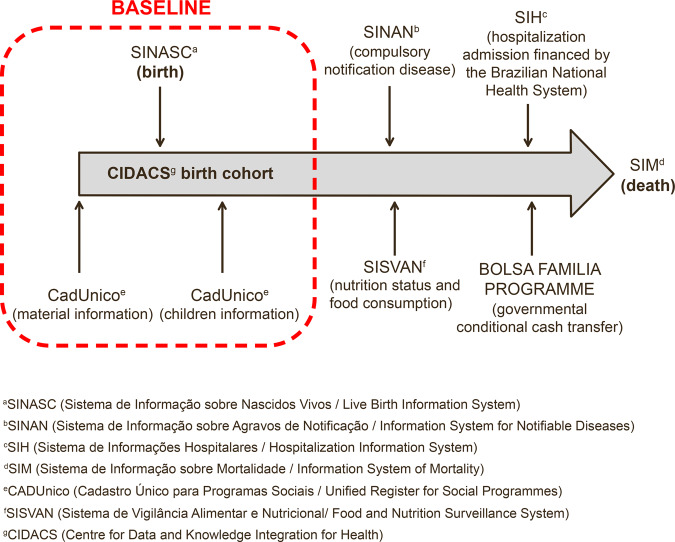
CIDACS birth cohort

The individuals included in CIDACS Birth Cohort will be dynamically followed from birth to death. Brazil has several mandatory national health and social registries that allow us to track a range of events throughout the individual’s life, including hospitalizations, infectious diseases occurrence, nutritional status, enrolment in social protection programmes and death (Figure 1). The follow-up will proceed using two linkage strategies: (i) deterministic linkage through unique national identification numbers that allow the cohort participants to be linked to periodically renewed socioeconomic information in CadUnico datasets (by CadUnico regulation, as long as the person is a candidate to receive or recipient of one of the several Brazilian government social protection programmes, they have to update the information every 2 years); (ii) non-deterministic linkage of the baseline of CIDACS Birth Cohort with health administrative datasets. The linkage to update the information will be done every 2 years.

The CIDACS Birth Cohort records are being linked with: SINAN [Sistema de Informação sobre Agravos de Notificação (Information System for Notifiable Diseases)]; SISVAN [Sistema de Vigilância Alimentar e Nutricional (Food and Nutrition Surveillance System)]; SIH [Sistema de Informações Hospitalares (Hospitalization Information System)]; and SIM [Sistema de Informação sobre Mortalidade (Information System of Mortality)]. The primary data available at each phase of the CIDACS Birth cohort are listed in Table 2.

**Table 2:** Dataset description, Centro de Integração de Dados e Conhecimentos para Saúde (CIDACS) Birth Cohort

Phase	Data source	Data collected from	Measurements	Sensitivity of the system	Period available
Baseline	SINASC (Sistema de Informação sobre Nascidos Vivos/Information System of Live Birth )	The form is completed by a health professional who was present at the delivery and records information about all births in Brazil	Characteristics of the newborn (sex, Apgar score 1 and 5 min, birthweight, presence of abnormality, congenital anomalies identified at birth using the ICD-10 code), identification of the place of birth, characteristics of the mother (name, age, marital status, education, race, place of residence), father's name and age, characteristics of pregnancy and delivery (number of previous pregnancies of live births, stillbirthsor abortions, length of gestation, type of delivery, number of fetuses, number of visits to prenatal care facilities, which health professionals were present at the delivery)	Records information of 97% of Brazilian live births	2001–15
Baseline	100 Million Brazilian Cohort baseline	The baseline of the 100 Million Brazilian Cohort was created using administrative records from over 114 million individuals aged 16 years or older, whose families applied for social assistance via the Cadastro Único para Programas Sociais (CadUnico)	Socioeconomic and demographic conditions (information on family dynamics, childcare arrangements, parental employment, income, housing, family formation and dissolution, social programmes information, household characteristics)	More than 50% of the Brazilian population	2001–15
Ongoing	SINAN (Sistema de Informação sobre Agravos de Notificação/Information System for Notifiable Diseases)	Suspected and/or confirmed cases of a list of infectious disease must be reported to the Epidemiological Surveillance service on a specific numerated notification form, which is available in any local health facility. This form can be filled in by any health professional who suspects disease. It is disease specific	Date of notification, date of onset of symptoms, name of the patient, date of birth, age, sex, whether pregnant or not, race, education, mother's name, municipality of residence, municipality of notification, address, if it is laboratory or epidemiological confirmation, and case evolution. There is specific information according with notified disease	Depends on disease	2002–15
Ongoing	SIM (Sistema de Informação sobre Mortalidade/Information System of Mortality)	This system uses the death certificate, a legal document that can only be completed by a physician, and records information about all deaths in Brazil	Differentiate between fetal and non-fetal deaths. Characteristics of the dead person (date of death and birth, name, name of the mother and father, sex, race, marital status, occupation and education, address), identification of the place of death (hospital, home, public place and includes the address of the place), characteristics of the mother (name, age, marital status, education, occupation, race, number of births, place of residence, length of gestation, number of previous stillbirths or abortions, type of delivery, number of fetuses in the current pregnancy), birthweight (this block should be filled only in the case of fetal deaths or infant mortality), cause of death using ICD-10 code	Varies by place, range 70–95% of Brazilian deaths	2001–15
Ongoing	SIH (Sistema de Informações Hospitalares/Hospitalization Information System)	All hospital admissions financed by the Brazilian National Health System	Patient personal information, date of hospitalization, duration, type of hospital, costs incurred, and causes of hospitalization	Around 70% of all hospitalizations in Brazil	2008–15
Ongoing	SISVAN (Sistema de Vigilância Alimentar e Nutricional/Food and Nutrition Surveillance System)	Anthropometric and food consumption data of SUS users are entered into the system by primary care workers through an online platform accessed at the health unit or department	Date of birth, age, sex, race/ethnicity, anthropometric data; anthropometric measurements collected include weight and height, birthweight for children under 2 years, waist circumference for adults, calf circumference for elderly, and pregestational weight for pregnant women. Breastfeeding practices, complementary feeding, eating behaviours and consumption of healthy and unhealthy foods	Varies according to target population. Around 30% among under-fives and 17% among pregnant women	2008–15

The Information System for Notifiable Diseases (SINAN) is the compulsory notification system for a list of infectious diseases, including dengue, zika, tuberculosis and chikungunya.^22^ Suspected and/or confirmed cases must be reported to the Epidemiological Surveillance Centre on a specific numbered notification form which is available in any local health facility. It collects information on the date of notification, date of onset of symptoms, date of birth, name of the patient, age, sex and address. The Epidemiological Surveillance Centre then investigates to confirm or discard the suspicion based on the Brazilian definition of case, specific for each disease. The quality of the data and years varies according to notified disease.^23^

The Food and Nutrition Surveillance System (SISVAN) will be used to assess child and maternal nutrition. Data from this system are available over 2008–15 and has information on anthropometric measurements, including weight and height, food consumption, breastfeeding and complementary feeding practices. The national population coverage of SISVAN ranges between 10% and 15%, mainly among children and adolescents. For those registered in the cash transfer programme Bolsa Familia, who are also enrolled in the CadUnico, the SISVAN coverage varies from 57% to 86%.^24^

All hospital admissions financed by the Brazilian National Health System (about 75% of all hospitalizations in Brazil) are recorded in the Information System of Hospitalizations (SIH). The hospitalization system includes personal patient information, date of hospitalization, duration, type of hospital, costs incurred, and causes of hospitalization.^25^

The Information System of Mortality (SIM) uses the death certificate, a legal document. This form collects information on the deceased individual and the conditions, place and cause of death. In the case of fetal deaths or infant mortality, it also includes maternal characteristics. In 2015, it was estimated that SIM registered more than 97% of the Brazilian deaths.^26^

## What has been measured?

The CIDACS Birth Cohort includes basic information on the mother (name, place of residence, age, marital status, education) and her obstetric history [whether she had a stillbirth or miscarriage, whether she had a previous caesarean section (CS) or vaginal delivery], the pregnancy (length of gestation, type of delivery, fetal presentation), the newborn (birthweight, presence of congenital anomalies) and the antenatal care (number of visits and when care started). In addition to birth and maternal information obtained from SINASC, socioeconomic and demographic data from the 100 Million Brazilian Cohort, such as information on family dynamics, child care arrangements, parental employment, income, housing, family formation and dissolution, are available in the baseline of the CIDACS Birth Cohort. Information on growth, breastfeeding and infectious disease has been included in the cohort follow-up. Although most variables have less than 10% missing data, there is a substantial proportion of missingness for the variables on the mother’s history of stillbirth or miscarriage (16% missing) and the employment situation of the household head (54% missing).

## What has it found?

To date, the CIDACS Birth Cohort has been used to analyse birth and mortality outcomes. Preterm births (<37 weeks of gestational age), low birthweight (<2500 g) and congenital anomalies were observed in 8.1%, 8.3% and 0.7% of the total births included in the cohort, respectively (Table 1). The deaths occurred from the first hours of life to the age of 14 years, and more than 80% of the deaths in our cohort occurred before the first year of life, mainly during the neonatal period (less than 28 days old).

Further linkage between CIDACS Birth Cohort baseline and other follow-up datasets are ongoing. The linkage with SINAN is being held to evaluate the impact of maternal infections, including zika and syphilis, on early outcomes (prematurity, low birthweight, congenital anomalies) and late outcomes (hospitalization and mortality). In addition, the linkage with SISVAN is being conducted in order to analyse child growth curves and the effect of maternal nutrition on birth and child growth outcomes.

## What are the main strengths and weaknesses?

CIDACS Birth Cohort has several strengths. First, it links health and social data coming from various government sectors, adding enormous value to already existing health data in determining both the drivers of health and the consequences of ill health. Second, its longitudinal structure makes possible to: (i) add new exposures or outcomes over time; and (ii) study outcomes at different times of exposure, including long-term outcome. Third, the large sample sizes allow analysis of small groups and rare events in ways that are not possible in projects that are dependent on the primary collection of new data. Fourth, we have included in our data information on isolated populations, such as Iindigenous people and Quilombo communities descended from AfricanBrazilian fugitive slaves. Fifth, the use of administrative data eliminates the risk of recall bias, which is a problem if data collection relies on self-reports of service use (e.g. hospitalization or birth). Sixth, the linkage has been conducted with robust and accurate software developed in-house (CIDACS-RL), and a specialized team evaluates each linkage performed at CIDACS.

There are some limitations that must be considered when analysing the CIDACS Birth Cohort. To measure follow-up can be a complex task in large, linked datasets where individuals have complex histories and errors can be present. The cohort baseline is the linked population of SINASC and population from the baseline of the 100 M Brazilian cohort, both routinely collected data that have not been designed for research purposes. Therefore, it brings well-known limitations relating to missing, underestimation and potential misclassification of data. For example in SINASC, the proportion of preterm births recorded was found to be underestimated by 15%, and misclassification, based on the criteria used to assess the gestational age at birth information (date of the last period), could have occurred.^27^ However, these errors probably affected the entire dataset. We have a considerable proportion of missing values in variables that are not mandatory in CadUnico, such as the occupation of the household members (54%). Nevertheless, the description of all individuals in the household (e.g. sex, age, education and ethnicity) and variables such as income, key variables that are used as eligibility criteria for social programmes, have good completeness.

A limitation that must be discussed concerning each specific research question is the characteristics of people enrolled at CadUnico (poorest half of the Brazilian population). There is a socioeconomic gradient that influences prevalence estimates, as reflected in higher rates of vaginal delivery that, in Brazil, is less common among wealthy families (Table 1). However, the CIDACS Birth Cohort aims to provide valid estimates of associations between putative causal factors and disease, and the prevalence of both exposures and diseases may be different from what is found in the general population. However, the estimate of association can still be valid. Several validity studies will be performed to address this question.

The linkage process posed several challenges, such as linking different individuals (mother-baby) due to the limited numbers of identifiers that have tended to yield higher rates of linkage error, commonly due to inaccurate or incomplete provision of identifiable data. The most critical barrier to linking maternal and live birth records is the limited availability of common and complete personal identifiers, which directly impacts on sensitivity results, that tend to be lower. A validation study estimated that the overall proportion of linked people between 2001 and 2015 was 59%. However, this was not constant over the years, and from 2012 the sensitivity reached about 80%, reaching values similar to studies developed in Georgia and New Jersey, USA.^28^^,^^29^

## Can I get hold of the data? Where can I find out more?

Data that support the information presented are available upon request from the CIDACS and on ethical approval. The data are not publicly available due to restrictions, as they contain information that could compromise the privacy of the research population.

Currently, only national and international researchers who collaborate with CIDACS, and authorized staff from government agencies, can have controlled access to de-identified linked data. These individuals and organizations must be committed to advancing scientific knowledge or generating evidence for public policy formulation. Researchers can access relevant de-identified data for their proposed study objectives exclusively via secure remote access to virtual machines.

Persons who wishes to receive authorization must: (i) be affiliated to the institution or be identified as collaborators; (ii) present a detailed research project together with ethical approval by an appropriate Brazilian institutional; (iii) provide a clear data plan restricted to the objectives of the proposed study and a summary of the analyses plan intended to guide the linkage and or data extraction of the relevant set of records and variables; (iv) sign terms of responsibility regarding the access and use of data; and (v) perform the analyses of datasets provided using the CIDACS data environment, a safe and secure infrastructure that provides remote access to de-identified datasets and analyses tools. For more information, please visit the CIDACS website [https://cidacs.bahia.fiocruz.br/] or contact us via email [cidacs@bahia.fiocruz.br].

**Profile in a nutshell**

- The Centro de Integração de Dados e Conhecimentos para Saúde (CIDACS) Birth Cohort is a Brazilian population-based cohort derived from linked national data, developed to investigate the relationships between prenatal and early life events, and health-related outcomes.
- The CIDACS Birth Cohort population is composed of 24 695 617 (55%) children born alive in Brazil between 1 January 2001 and 31 December 2015, which linked with the baseline of the 100 Million Brazilian Cohort through maternal information. We have information on children from birth up to 14 years of life.
- Linkage was performed using a record linkage tool (CIDACS-RL) using maternal name, municipality and date of birth records or age. The link between the composite file SINASC-100 Million Brazilian Cohort with other Brazilian datasets (mortality, hospitalization, infectious disease and nutrition status) will provide the cohort follow-up and outcomes information.
- Currently, national and international researchers who collaborate with CIDACS have controlled access to de-identified linked data. Qualified investigators who wish to receive authorization to access the data must present a detailed research project together with ethical approval, provide a plan of analyses and sign terms of responsibility.

## Funding

CIDACS received core support from the: Health Surveillance Secretary, Ministry of Health, Brazil; Fundação de Amparo à Pesquisa do Estado da Bahia (FAPESB); Wellcome Trust (grant number 202912/Z/16/Z); Financiadora de Estudos e Projetos-FINEP; and Secretary of Science and Technology of the State of Bahia-SECTI. E.S.P. is a fellow supported by the Wellcome Trust (grant number 213589/Z/18/Z). The funders had no role in study design, data collection, data analysis, data interpretation or writing of the report.
